# Edinburgh Surgical Hospital

**Published:** 1831

**Authors:** 


### XXIX.

### EDINBURGH SURGICAL HOSPITAL:

#### 1. REUNION OF FRACTURED BONES.*

MR. SYME continues to report from this his own hospital. We have on several occasions had occasion to praise and to blame these reports; to praise them as evincing assiduity and industry, to blame them for a spirit which, if not egotistical, might fairly give a colour to such an imputation. We know that it is the custom of most hospitals supported by voluntary contributions, to publish an annual or semi-annual report, setting forth their advantages to the community, their lists of patients relieved and cured. This is looked on with charitable feelings, and if a slight disposition to embellishment is noticed, it is not visited by any severity of criticism. But such a taint in a document intended for the profession, as a medical report in a medical journal undoubtedly must be, is not calculated to enhance nor even give their proper degree of value to its merits. We do not affirm that Mr. Syme's reports are characterized by this failing; we merely hint, that a superficial reader might be tempted to think them so. When Mr. S. informs us that, ever since the institution of the hospital, "it has been greatly resorted to for relief in cases of fracture, both by in and out patients," we cannot doubt that, when its merits become still more generally known than they now are, persons will voluntarily submit to fractures in order to partake of them. In this town individuals with broken legs are usually carried to the nearest hospital, and comparatively seldom *resort* of themselves to any one in particular. But probably no Metropolitan institution has acquired the deserved celebrity of Mr. Syme's. In the Edinburgh hospital, then, there have been no less than 17 cases of fracture during the last month, "when, it is true such accidents most abound, from the combined effect of intemperance and frosty weather." Of this long list two were fractures of the clavicle, three of the humerus, and two of the metatarsus and metacarpus. The remainder were fractures of the lower jaw, femur, patella, and tibia and fibula. We conceive that there are few of the larger London hospitals but would furnish as great a list in a fortnight or even in a week.

It is not our intention to notice this report at any length. Our readers are aware that many different accounts of the mode of re-union of broken bones have at various times been given to the world. The experiments of Dupuytren and Breschet, and of Mr. Howship have thrown much light upon the process, and established the important part which is played by the periosteum and soft parts. The following cases are valuable as bringing from human dissections proofs of the correctness of deductions from experiments on the lower animals.

*Case* 1. A woman, æt. 52, was ad-

* Ed. Med. and Surg. Journ. No. 107.

mitted with fracture of the right thigh-bone in its lower third. Pasteboard splints and the long splint of Dessault were applied, and she seemed to be doing well till the 11th day, when symptoms of fever came on, and on the 14th day she died apparently with œdema of the glottis.

"On dissection, the fracture was found to extend obliquely from near the middle of the bone down to the external condyle. The muscular fibres and cellular substance in the neighbourhood of the injury were altered in colour as well as consistence, by the effusion of gelatinous matter into their texture. A kind of bag or capsule was thus formed, embracing the whole extent of broken surfaces, and containing two or three ounces of fluid blood. The parietes composing it were in some parts connected to the very edge of the bone, but in others they became adherent to it at a distance of an inch or more from the extremity, leaving a space to this extent uncovered, and apparently denuded of periosteum. When carefully examined, this exposed portion was ascertained to be covered by a thin layer of gelatinous substance, which did not possess the toughness or other characters of a membrane; and the respective surfaces of the bone had a covering of the same kind. The medullary membrane was very vascular, and more distended than usual.

In examining the structure of this bag, I endeavoured to ascertain which of the natural tissues entered into its formation, and in what parts of it, if any, ossification had commenced. On tracing the periosteum from the sound bone, I found that where the bag adhered, that membrane became thick, and evidently continuous with its walls. It seemed probable that where the membrane had been stripped off the bone, as already mentioned, it might assist to form, in some small part, the sac in question; the great extent of which, however, was evidently constituted by the neighbouring tissues, whatever they happened to be, muscle, tendon, fat, or cellular substance, all being reduced to the same appearance internally, by vascularity of the surface, and the same consistence, by the interstitial effusion of organizable matter.

On introducing my finger into the bag, so as to feel if there were any indications of ossification, I perceived some small grains or specks of bone, which, when minutely examined, presented a stellated appearance, and were ascertained to lie in the substance of the capsular membrane. When examined in the same way near its connexion with the bone, it was found to contain much larger masses possessing osseous firmness; in order to ascertain the precise seat and origin of which, I carefully dissected the membrane where they existed, and then found that they lay completely imbedded within it, having a covering from it on both sides; also that they did not adhere to the bone, being separated from it by a thin layer of the membrane, so as to admit of a slight degree of motion; but at these parts, the shaft itself had begun to shoot out a growth of new bone."

*Case* 2. An old woman, æt. 70, was admitted with compound fracture of the left leg, close to the ankle. In the course of a month the cure seemed to be complete, and ten days after that the woman was dismissed with a perfectly straight limb. In the course of ten days from this time she died, and Mr. Syme was enabled to examine the bones.

"When divested of their muscular coverings, they presented an appearance hardly differing from that naturally belonging to them. All the pieces into which they had been broken were firmly united to each other and to their shafts, and were covered with a periosteum of usual consistence. On closer examination, the interstices between these portions were found to be occupied by a soft bloody gelatinous substance, to ascertain the precise extent of which, the preparation was macerated. When all the interstitial matter had been thus separated, it was seen that the united fragments of the tibia, which were thirteen in number, constituted merely a skeleton, so to speak, of the cylinder, and that the central cavity remained entirely vacant. On examining the internal surface of this imperfect shell, it was evident that an ossific process had been going on over the whole of it, and I have no doubt, that, if the patient had lived some months longer, the bones would have become completely solid. The fibula presented similar appearances, though on a smaller scale, and the process of reunion was more nearly perfected. There is in my possession the preparation of a thigh-bone which was fractured through the neck and trochanters, and was treated by my friend Mr. George White. The patient died two months after the accident from some other cause. It now appears, the bone having been macerated, that all the broken portions are firmly united together at the edges, but that all their internal surfaces remain perfectly distinct and separate. The appearance, in short, is very nearly the same, and, I believe, would also have terminated in compact ossification, if the necessary time had been afforded."

### II. URINARY CALCULUS.

The following fact may be useful to others; it appears to have been instructive to Mr. Syme. Mr. S. extracted two large stones from a sac formed by dilatation of the membranous part of the urethra. Some uneasiness continued after the operation, which was referred to a stricture that had previously existed, but was not relieved as the stricture was dilated. Mr. S. now passed an instrument into the bladder, and found a stone in it. The parents would not permit him to remove it, but Mr. Syme has since heard that the calculus has been extracted by another surgeon. In short, by this little lapsus Mr. Syme lost his patient when he found the stone. Other surgeons may take the hint.

In similar cases Mr. Syme recommends us to divide the prostate, so as to admit the finger, and to make a complete scrutiny into every corner of the bladder. Thus a boy, æt. 3, was admitted with symptoms of stone. On introducing a sound, a calculus was felt lying in the prostatic part of the urethra, and it was removed. Mr. S. dilated the neck of the bladder with a bistoury, and introduced his finger so as to ascertain positively that there was no concretion in the bladder. The boy was dismissed cured in twelve days.

Mr. Syme relates a curious case in which he performed the operation of lithotomy. The man had suffered excessively from the symptoms of stone for nearly three years, and he was accustomed to take as much as sixty-two grains of opium for his daily dose. Mr. S. performed the operation, and for the first six days the patient did well, taking from six to eight hundred drops of laudanum daily. On the sixth day, however, it was thought better to begin to diminish the quantity of laudanum, and in the evening he complained of exhaustion and general uneasiness, the pulse having risen to 80, the tongue dry. Laudanum was prescribed, and next day he was rather better, but had a little mucous rattle in the chest. A little antimonial wine was added to the laudanum, and a blister applied. During the three next days he became progressively worse, another blister was applied, and on the third day he complained of violent pain in the left lumbar region. This continued till his death, on the following day. On dissection the lungs were found gorged with mucus; the descending colon was found extremely contracted, and these were all the morbid appearances. Mr. S. can say little explanatory of the case; neither can we.

In a case of retention of urine from a calculus at the bulb of the urethra, which could be felt through the integuments a little behind the scrotum, Mr. Syme adopted the following measures. He first dilated the passage after the manner of the Egyptians, that is, by blowing into it with a tube, having previously, to facilitate the passage of the stone, introduced a little oil. Then making pressure behind the calculus, he brought it forward to the anterior part of the scrotum, and, not being able to advance it further, he cut down upon it in that situation, and removed it. The boy was well in two or three days. In this patient Mr. Syme would not operate while the stone was posterior to the scrotum, "because the parts are thick, and from their laxity favourable to the effusion both of blood and urine." Now it is a singular circumstance that Mr. Brodie has come to a diametrically opposite conclusion. Having removed several calculi by cutting into the urethra behind the scrotum with success, he was induced in one instance to bring the stone by the urethra-forceps anterior to the scrotum, and cut down upon it in that situation. The urine afterwards gravitated into the scrotum, troublesome abscesses succeeded, and from that time Mr. Brodie has always preferred cutting on the calculus behind the scrotum. By the way, we should imagine that a pair of urethra-forceps would have been much superior to the clumsy, albeit Egyptian, method of blowing up the canal.

### III. CANCEROUS ULCERATIONS OF THE FACE.

Ulcers of the lip are sometimes prevented healing merely by the motion of the part, or the indirect irritation produced by disorder of the digestive organs, when pressure and a sulphate of zinc or black-wash is sufficient for the cure. Thus a farmer applied to Mr. Syme with a small superficial ulcer on the lip, nearly the size of a sixpence, which had resisted all the means already employed. Under the application of a piece of lint dipped in black-wash, covered by a piece of oil-silk, and supported by a bandage to prevent motion of the lip, the sore was diminished to half its former size in two or three days, and the farmer went into the country.

For cancerous affections of the tongue Mr. S. recommends the knife in preference to the ligature. Having removed a large ulcerated tumour of the tongue, nearly half of which was engaged in the disease, the wound healed, and all seemed doing well. Not long afterwards the complaint began to return, affecting not only the tongue but the floor of the mouth.

